# Population’s health information-seeking behaviors and geographic variations of stroke in Malaysia: an ecological correlation and time series study

**DOI:** 10.1038/s41598-020-68335-1

**Published:** 2020-07-09

**Authors:** Kurubaran Ganasegeran, Alan Swee Hock Ch’ng, Zariah Abdul Aziz, Irene Looi

**Affiliations:** 1 Clinical Research Center, Seberang Jaya Hospital, Ministry of Health Malaysia, Penang, Malaysia; 2 Department of Medicine, Seberang Jaya Hospital, Penang, Malaysia; 3 Clinical Research Centre, Sultanah Nur Zahirah Hospital, Ministry of Health Malaysia, Terengganu, Malaysia; 4 Medical Department, Sultanah Nur Zahirah Hospital, Terengganu, Malaysia

**Keywords:** Medical research, Epidemiology, Outcomes research

## Abstract

Stroke has emerged as a major public health concern in Malaysia. We aimed to determine the trends and temporal associations of real-time health information-seeking behaviors (HISB) and stroke incidences in Malaysia. We conducted a countrywide ecological correlation and time series study using novel internet multi-timeline data stream of 6,282 hit searches and conventional surveillance data of 14,396 stroke cases. We searched popular search terms related to stroke in Google Trends between January 2004 and March 2019. We explored trends by comparing average relative search volumes (RSVs) by month and weather through linear regression bootstrapping methods. Geographical variations between regions and states were determined through spatial analytics. Ecological correlation analysis between RSVs and stroke incidences was determined via Pearson’s correlations. Forecasted model was yielded through exponential smoothing. HISB showed both cyclical and seasonal patterns. Average RSV was significantly higher during Northeast Monsoon when compared to Southwest Monsoon (P < 0.001). “Red alerts” were found in specific regions and states. Significant correlations existed within stroke related queries and actual stroke cases. Forecasted model showed that as HISB continue to rise, stroke incidence may decrease or reach a plateau. The results have provided valuable insights for immediate public health policy interventions.

## Introduction

Conventional epidemiological data resources such as disease registries or national health and morbidity surveys that explore temporal or geographical variations across populations are often dependent on large scale community-based surveillance studies. These data are regarded as the “gold-standard” for epidemiology as they are capable of yielding observations based on geographical gradients or stratifications by age or gender^1^. Such data resources are retrospective in nature, resource intensive and have lag periods of data availability^2^, limiting capabilities for urgent analytical inferences or evidence synthesis for public health policy implementations. Another limitation of such conventional approaches is the inability to monitor real-time population’s health information-seeking behaviors (HISB) on emerging threats of diseases.

With the rise of Health Web 2.0, Population Health Data Science (PHDS) has emerged as an art of science that transforms real-time data into actionable knowledge that informs, influences and optimizes decision making promptly^3^. PHDS integrates public health medicine, robust medical statistics, health and behavioral sciences within human centered designs for knowledge integration^3^. The adoption of PHDS connotes the era of information overload within big health data, allowing real-time HISB analysis of stroke to be conducted through borderless internet connectivity. Within these applications, Google Trends has been regarded as the best analyzer for real-time HISB analysis^4^.

Digital footprints left by online internet users potentially serve as proxies for monitoring disease activities and HISB at the community level, capable of providing real-time valuable insights into temporal and spatial trends of diseases being studied^5^. The bulk of literature has explored online HISB for a variety of diseases. These include neurological disorders such as multiple sclerosis^6^ and status epilepticus^7^, rheumatic diseases like systemic lupus erythematous (SLE)^8^, mental health conditions like suicidal thoughts^9^ and non-suicidal self-injury^10^, non-communicable diseases and risk factors such as cardiovascular disorders^2^, cancer^11^ and non-cigarette tobacco use^12^, and infectious diseases like AIDS^13^, Ebola^14^ and influenza^15,16^. The monitoring and analysis of internet data is conceptualized as infodemiology, providing real-time data, tackling time lag for data analysis and forecasting of disease patterns.

Studies conducted till date have mostly used and analyzed single datasets of either internet multi-timeline data or conventional surveillance data (e.g. disease registries) separately, limiting the potentials to explore correlations with real-time HISB and incidence of diseases. The epidemiological trends of stroke occurrences across populations have been influenced by geographic variations, demographics and socio-economic attributes^1^. These trends were speculated to be influenced by weather, temperature or seasonal variations in some studies^17–19^. The current study was the first in Asia, from the Malaysian perspective that aimed to determine the trends, correlations, weather and geographic variations of stroke, and to subsequently yield a forecasted model of real-time HISB and stroke incidences in the country for the next 3 years.

## Review of literature

### Global epidemiology of stroke

Stroke is one of the leading causes of mortality and disability worldwide^20,21^. In 2016, stroke accounted for 116.4 million disability-adjusted life years (DALYs) and 5.5 million deaths globally^22^. There were approximately 80.1 million stroke cases reported in 2016 that afflicted 41.1 million women and 39 million men respectively^22^. Between 1990 and 2017, there was an 11.3% decrease in age-standardized stroke incidence rate worldwide (150.5 per 100,000 people in 2017)^23^. But this scenario was accompanied by an overall 3.1% increase in age-standardized stroke prevalence rate (1,300.6 per 100,000 people in 2017), with 33.4% decrease in age-standardized stroke mortality rate (80.5 per 100,000 people in 2017) in the same period of time^23^. Escalated trends of age-standardized stroke incidence rates were observed mostly in middle-income countries^23^. Regional differences found that the incidence of stroke was the highest in East Asia, followed by the Eastern European region and the lowest in Central Latin America^24^.

### Stroke as a public health issue in Malaysia

The epidemiological literature of stroke in Malaysia was scarce until the implementation of the National Neurology Registry (NNEUR) of Malaysia in 2009^25,26^. Malaysia witnessed an escalating incidence of stroke cases, being the third most common cause of mortality and topped the nation’s disability rate^27^. In 2016 alone, stroke accounted for 11,284 cases, mostly affecting men (55%) and those aged 60 years or older (60%)^26^. Age-standardized stroke mortality rates were 103 per 100,000 in men and 97 per 100,000 in women^25^. Significant functional disabilities and psychiatric morbidities posed substantial burden to patients, caregivers, healthcare systems and providers^25^, thus escalating high economic burden^28^.

### Google Trends related studies

Google Trends has been valuable to explore trends, seasonality and correlations for a variety of neurological and non-communicable diseases. Walcott et al^29^ used Google data to determine the prevalence of stroke in the USA. They found that disease-specific search queries related to stroke correlated well with geographical differences across states and the correlation model provided a metric to evaluate health disparities^29^. Senecal et al^30^ hypothesized the importance of online search symptoms for early identification of cardiovascular diseases. They found correlation of online symptom of chest pain with coronary heart disease epidemiology^30^. Kumar et al^2^ were eager to determine if temporal and geographical interests in seeking cardiovascular disease (CVD) information online would follow a seasonal or geographical pattern similar to those observed in real-world data. They performed an ecological correlation study by using online search queries from Google Trends and age-adjusted estimates of mortality associated with heart disease, heart failure and stroke per 100,000 persons. They found that query volumes followed strong seasonal patterns and yielded moderate to strong positive correlations between state-level search query volumes and mortality rates^2^. Bragazzi^6^ explored internet usage data for seeking health materials for self-care and self-management purposes in monitoring multiple sclerosis using Google Trends. The study concluded that Google Trends was a reliable tool for monitoring multiple sclerosis with significant correlations found between clinical manifestations and treatment across different states in Italy^6^.

## Motivations of the current study

As conventional epidemiological data collection and analysis is labor intensive and time consuming, Google Trends has offered an alternative to provide real-time data. Such alternatives, being part of PHDS has given an opportunity to public health advocates to yield immediate evidence for crafting disease control and prevention strategies. The diversity of subjects that Google Trends could explore for examining changes in search interest overtime and the usefulness of this tool in assessing human behavior is evident that online search traffic data analytics being correlated with conventional epidemiological data will be valuable to explore, predict and forecast health behavioral changes amongst populations^4^. Given the high prevalence of stroke in Malaysia in recent years, it is timely to offer this novel epidemiological surveillance data analytics tool at the population level for faster evidence synthesis.

## Methods

### Study population and design

This countrywide ecological correlation and time series study was conducted between January 2004 to March 2019 by employing digital and spatial epidemiological analytics for the study of stroke HISB and incidence of stroke among the Malaysian population. Digital epidemiology adopted concepts of “infodemiology” and “infoveillance” that was recently coined as the “new public health” to study online HISB of health related conditions and disease patterns, distributions, trends, variations, and correlations by using novel internet data streams^31^. While “infodemiology” has been defined as the science of distribution and determinants of information in an electronic medium, specifically the internet (Google Trends) with the ultimate aim to inform public health policy, “infoveillance” has been conceptualized as the longitudinal tracking of “infodemiology” metrics for surveillance and trend analysis. Spatial epidemiological analytics that utilized geographic information systems (GIS) was employed to understand the distribution of HISB and stroke incidence across regions, cities and states in Malaysia.

### Data source

Online HISB of stroke was retrieved from Google Trends multi-timeline search queries data. Google Trends, an online tracking system of internet search volumes that merged with Google Insights for Search (Google Inc.)^32^, was searched between years 2004 until 31st March 2019 for the terms “stroke,” “strok (Malay),” “angin-ahmar (Malay),” “cerebrovascular accident,” and “CVA” in Malaysia. Related domains of “stroke and organ affected,” “stroke types,” ‘stroke symptoms,” “stroke signs,” “stroke risk factors,” “stroke treatment” and “stroke prevention” were also explored. Google Trends automates normalized data for the overall number of searches and provides values as relative search volumes (on a scale from 0 to 100; value 0 does not necessarily indicate no searches, but rather indicates very low amount of search volumes that are not included in the results) in order to compare variations of different search terms across geographical settings and periods. This approach has been applied and validated. All queries and search volumes related to stroke were downloaded via *.csv file* format.

Conventional surveillance data of actual stroke counts in the country was obtained from the NNEUR, a prospective, multicenter hospital-based registry that captures data of acute stroke patients admitted across Ministry of Health Malaysia hospitals nationwide. The registry is an on-going effort funded by the government of Malaysia and consists of fifteen participating stroke hospitals across the Peninsular Malaysia and Borneo region. The registry aims to capture a comprehensive epidemiological surveillance data of stroke in the country. NNEUR participating stroke hospitals enroll confirmed hospitalized stroke patients within two weeks of symptoms onset^26, 33^. Actual stroke counts that were available between 2012 and March 2019 across states were retrieved and tabulated.

### Procedure

The procedure of data retrieval, exploration and analysis was conducted based on the validated methodological framework proposed by Mavragani and Ochoa^34^. It includes four major steps as follows:

I. *Step 1: Measurement of online search interests (data overview)* We explore online interest for different terms or keywords (up to five) in the same region for the same period such as “stroke,” “strok,” “angin ahmar,” “CVA,” and “cerebrovascular accident” in Malaysia from January 01, 2004, to March 31, 2019. Related domains of stroke were also explored. As our search terms may encounter misspellings in English but correct in Malay (for e.g. “stroke” in English, but “strok” in Malay is equally correct for the language, but considered misspelled in English), we utilized the “+ feature during searches to aggregate the result volumes without eliminating it.
II. *Step 2: Explore seasonality or variations* This step aimed to detect variations or seasonality of web-based interest. It forms the platform if the data is suitable to proceed on examining relations between online search interests and actual events or disease cases.
III. *Step 3: Finding correlations* This step correlates web-based queries among them or with official actual data cases. The official actual stroke count data in Malaysia was obtained from the NNEUR.
IV. *Step 4: Predict and forecast* This final step aimed to predict and forecast stroke HISB with future incidence of stroke.

### Statistical methods

Statistical analysis was conducted using R version 3.5.1^35^ and IBM SPSS Statistics version 22.0^36^. We conducted time series analytics to explore trends of HISB of stroke in Malaysia. Seasonality over time, month and weather variations, coupled with top search queries and flux volumes was determined through Google Trends multi-timeline data. To test for differences in mean search volumes across weather and month, we used linear regression analysis with season or month as a categorical predictor, with the 95% CIs for percentage change being bootstrapped with 1,000 random samples.

Correlograms to check for autocorrelation and adjusted partial autocorrelation significance for time series was determined using Wessa Time Series^37^. In addition, we determined randomness of data through series of point time lags that reached zero or near-zero in yielded correlograms. The degradation of points to near zero, either rapidly or slowly determines stationary or non-stationary of the data in the correlograms.

Spatial epidemiology of choropleth maps were yielded through merged data from the Global Administrative Database (GADM-Level 1 Data—Malaysia) that was available from the Center of Spatial Sciences^38^. A list of stroke attributes and related terms of their flux volumes were correlated with their hit search data using Pearson’s correlation coefficient analysis. Pearson’s correlation analysis is the measure of linear correlation between two continuous variables^39,40^; in this study “stroke” search term as the dependent variable and stroke-related terms as independent variables retrieved from Google Trends search queries. The analysis yields Pearson’s correlation coefficient *(r)* and ranges between − 1 and 1^39,40^. A correlation of − 1 indicates that the two variables are negatively linearly related, a correlation of 0 means that the two variables do not have any linear relations, while a correlation coefficient of 1 means that two variables are perfectly positively linearly related^40,41^. Consistent with these statistical theories, we followed trends of recent time series studies that utilized Google Trends to explore correlations within search terms or between search terms and counts data of different diseases by employing Pearson’s correlation analyses^2,7,8,10^.

Subsequently, we performed an ecological correlation analysis^42,43^ to test whether search volumes were correlated with the actual incidence of stroke at state and country level using Pearson’s correlation coefficient analysis. Significance level was set at two tails (P < 0.05).

Finally, we forecasted a predictive model using exponential smoothing of Winters additive method to yield Malaysia’s Stroke 2.0, that aims to forecast HISB and projected incidence of stroke within the next 3 years. Forecasting and modelling methods in principle have two general approaches—exponential smoothing or moving averages^44^. On what determines the usability on one of those two approaches are the conditions of stationary and seasonality of the time series data^44–46^. Moving averages are highly appreciable in stationary time series^44^. As our time series data showed seasonality trends and was non-stationary, we opted for exponential smoothing^44^. Literature has identified that Holt-Winters exponential smoothing (a stochastic procedure of observations during the time) is better and more widely used due to its flexibility in seasonal variations^45,47^. The method assigns exponentially increasing weights when previous observations get closer to the current state, with older observations being assigned a relatively lesser weights^47^. Winters method offers two methodologies to execute forecasting analysis; either additive method or multiplicative method^45,46,48,49^. Additive method is used when the data shows seasonality that is roughly constant, while multiplicative method is used when seasonal variations change proportionally and rapidly to the level of time series^44–46^. As our data is more inclined to the former, we used the additive method. The mathematical formula is given below:

1 $$Level:\;S_{t} = \alpha \left( {\frac{{X_{t} }}{{I_{t - s} }}} \right) + \left( {1 - \alpha } \right) \left( {S_{t - 1} + T_{t - 1} } \right)$$

2 $$Trend:\;T_{t} = \gamma \left( {S_{t} - S_{t - 1} } \right) + \left( {1 - \gamma } \right)T_{t - 1}$$

3 $$Seasonality:\;I_{t} = \delta \left( {\frac{{X_{t} }}{{S_{t} }}} \right) + \left( {1 - \delta } \right)I_{t - 1}$$

4 $$Forecasting:\;\hat{X}_{t} \left( k \right) = \left( {S_{t} + kT_{t} } \right)I_{t - s + k}$$

in which α, γ and δ denote smoothing parameters, and *S*_*t*_*, T*_*t*_ and *I*_*t*_ represent smoothing equations of levels, trends and seasonality. The data from observed values (X_t_) is projected through the forecasting Eq. (4), at k steps ahead to yield prediction, $\hat{X}_{t} \left( k \right)$^46,50^.

### Ethics statement

This study was approved and registered with the National Medical Research Registry of Malaysia (registration number: NMRR-19-1067-48224-IIR).

### Conference presentation

Findings from this study was presented at the 6th Asia-Pacific Conference on Public Health, 22nd–25th July, 2019 at the Equatorial Hotel, Penang, Malaysia.

## Results

### Trends of stroke health information-seeking behaviors

**Figure 1:**
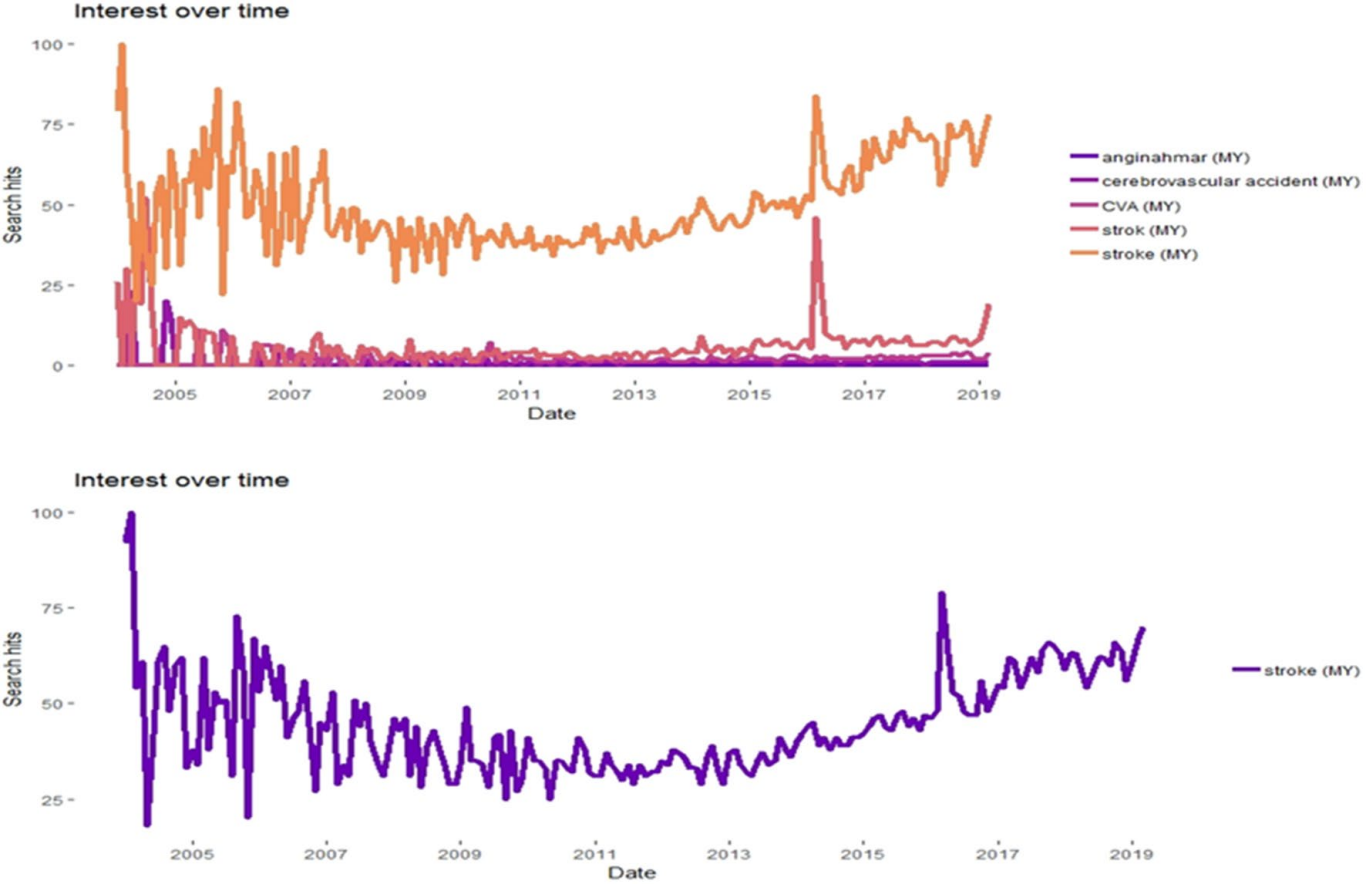
Google Trends of ‘stroke’ hit searches over the years. Data was mined since inception from 2004 till 31st March 2019. The top figure panel exhibits query patterns of all terms with similar meaning used in Malaysia: ‘stroke’ in English; ‘strok’ and ‘angin ahmar’ in Malay; ‘cerebrovascular accident’ and ‘CVA’ as medical terms. The bottom figure panel exhibits pattern of the most common search query, ‘stroke’ in English. Figure panels were created in R version 3.5.1^35^ (www.R-project.org).

**Figure 2:**
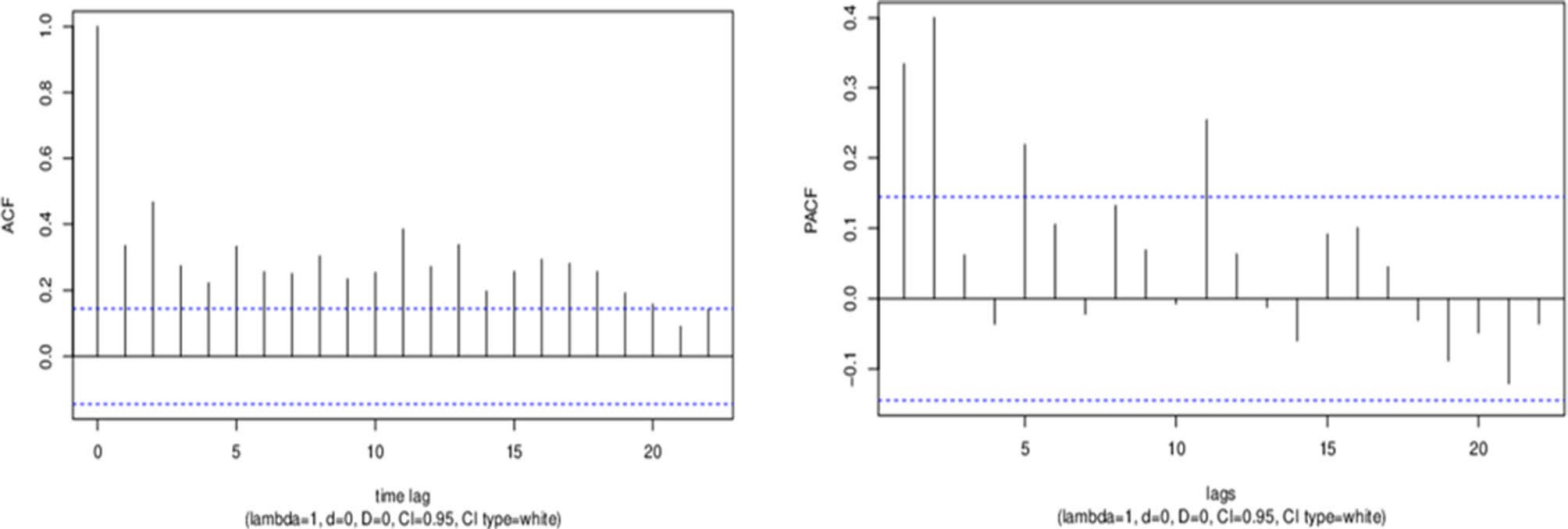
Autocorrelation and partial autocorrelation plots for ‘stroke’ search queries. Data was mined since inception from 2004 till 31st March 2019. Statistical significance exists between series of time lags (P < 0.05). Correlograms were plotted using wessa.net time series function^37^. Yielded parameters: lambda = 1, d = 0, and D = 0 indicated no transformation or differencing was applied before PACF was computed. 95% confidence interval (CI) was computed assuming white noise time series. *ACF* autocorrelation function; *PACF* partial autocorrelation function.

The most common search query was the English term ‘stroke.’ Between January 2004 and 31st March 2019 (n = 183), a total of 6,282 ‘stroke’ hit search queries were generated through Google Trends in Malaysia. The interest over time of internet search queries showed a cyclical pattern within a 2-year interval, and subsequently exhibited seasonality over the years (Fig. 1). Correlograms that yielded autocorrelation and partial autocorrelation plots showed statistical significance with series of time lags, and dataset was at randomness (Fig. 2).

### Variations of search volumes by months and weather

The mean percentage of stroke search volume was significantly higher for the period of January to April and June to December in comparison to the month of May (P < 0.01 for January–February, April, June–October and December compared to May; P = 0.016 for March vs May; P = 0.014 for November vs May) (Table 1). When analyzed by weather, average search volume was higher during the Northeast Monsoon in comparison to the Southwest Monsoon (P < 0.001) (Table 1).

**Table 1:** Mean percentage of stroke search volumes compared with reference month and weather.

	Mean percentage of Malaysia’s stroke search volume (95% CI)*	P-value
**Month**		
January	33.28 (29.28–37.28)	0.008
February	36.14 (31.14–41.71)	0.008
March	38.57 (29.28–48.14)	0.016
April	38.00 (31.62–44.08)	0.002
May	Reference	Reference
June	35.14 (29.57–40.56)	0.001
July	35.42 (30.85–40.14)	0.003
August	33.85 (27.13–40.57)	0.000
September	35.42 (30.22–41.00)	0.006
October	36.71 (31.00–42.71)	0.005
November	38.14 (32.42–44.71)	0.014
December	34.85 (28.71–41.42)	0.003
**Weather**		
Southwest Monsoon (May–September)	Reference	Reference
Northeast Monsoon (November–March)	181.0 (148.0–220.7)	< 0.001

Category with the lowest mean value was chosen as ‘reference.’

*Denotes bias corrected accelerated 95% confidence interval (95% CI).

### Geographic variations of stroke health information-seeking behaviors in Malaysia

**Figure 3:**
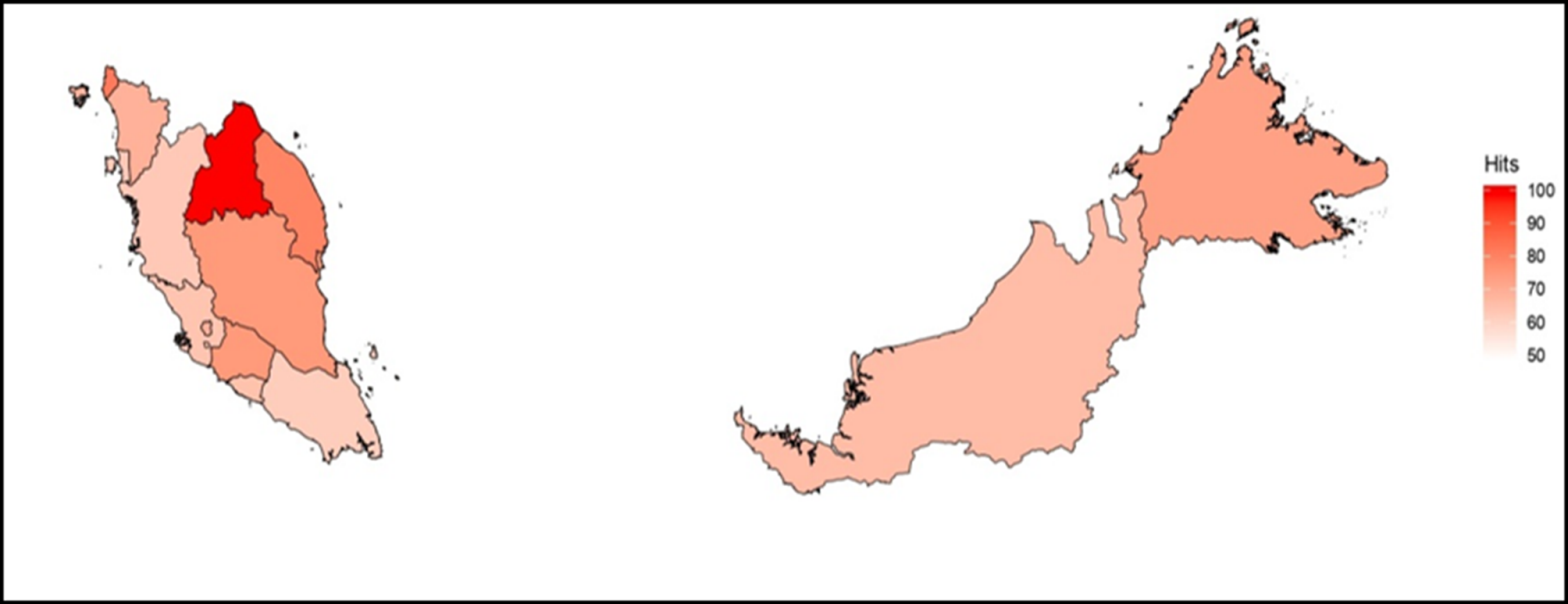
Choropleth map showing distribution of “stroke” search queries in Malaysia. Data was mined since inception from 2004 till 31st March 2019. Choropleth map was generated by merging Google Trends ‘stroke’ hit search queries multi-timeline data with the Global Administrative Dataset (GADM—level 1 data: Malaysia)^38^; available from the Center of Spatial Sciences at the following link: https://gadm.org/download_country_v3.html. Choropleth map was created in R version 3.5.1^35^ (www.R-project.org).

Figure 3 illustrates a choropleth map that exhibits the geo-spatial distribution of ‘stroke’ HISB across all states in Malaysia. The yielded map observed a geographical gradient within Peninsular Malaysia, with higher hit-search flux volumes originated from the East Coast Region (Kelantan and Terengganu), Northern Region (Perlis) and the Southern Region (Negeri Sembilan). The states from the Central Region (Selangor and the Federal Territories) yielded a relatively moderate to mild flux volumes. However, flux volumes from East Malaysia (Borneo states) were relatively moderate to high. The top five Malaysian states with high search flux volumes of ‘stroke’ were Kelantan (100), Perlis (83), Terengganu (81), Negeri Sembilan (76) and Pahang (76). The top five Malaysian cities or towns with high search flux volumes were Kota Bharu (100), Batu Pahat (82), Ampang Jaya (78), Kuala Terengganu (77) and Sungai Petani (76). Queries of ‘stroke’ search flux volumes were normalized, eliminating crude absolute values.

### Distribution of stroke in Malaysia

**Figure 4:**
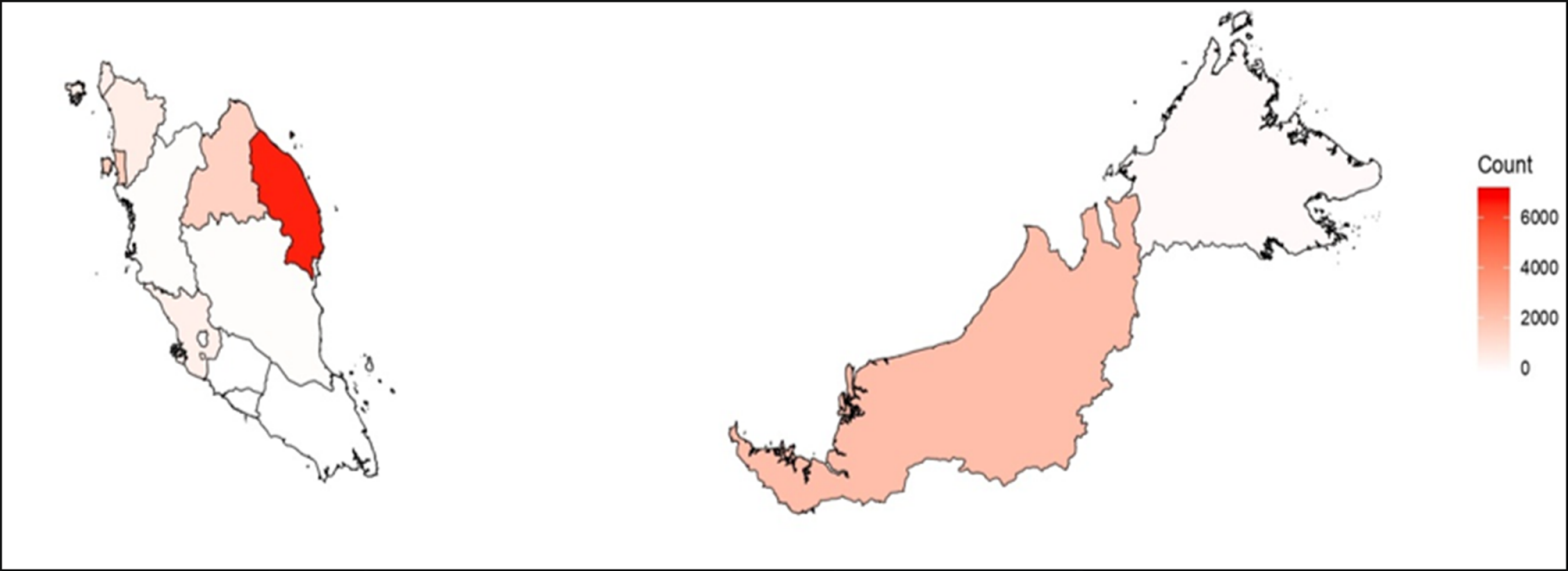
Choropleth map showing distribution of stroke in each state in Malaysia. Data was mined since inception from 2012 till 31st March 2019. Official count data was retrieved with permissions from the NNEUR of Malaysia – an official registry that captures stroke data within the Ministry of Health Malaysia facilities countrywide. Malaysia’s stroke count data included eleven states (excluded Federal Territories, Negeri Sembilan and Melaka due to unavailability of data for inclusion into analysis). Choropleth map was generated by merging actual counts data from the official NNEUR data with the Global Administrative Dataset (GADM – level 1 data: Malaysia)^38^; available from the Center of Spatial Sciences at the following link: https://gadm.org/download_country_v3.html. Choropleth map was created in R version 3.5.1^35^ (www.R-project.org).

Between 2012 and March 2019, there were 14,396 stroke cases recorded across eleven states in Malaysia. Within months, January recorded 1,351 cases, February (1,111 cases), March (1,296 cases), April (1,180 cases), May (1,305 cases), June (1,295 cases), July (1,054 cases), August (1,179 cases), September (1,045 cases), October (1,183 cases), November (1,311 cases), December (1,086 cases). Figure 4 exhibits a choropleth map that yields the geo-spatial distribution of ‘stroke’ cases in Malaysia. The generated map showed consistencies of geographical gradient between stroke cases and hit searches across regions within Peninsular Malaysia. Stroke cases were higher in the East Coast Region (Kelantan and Terengganu), Northern Region (Pulau Pinang, Kedah and Perlis) and the Southern Region (Negeri Sembilan). However, geographical gradient of stroke cases across states were contrary to hits search volumes, with Terengganu recorded a “red alert” of the highest stroke counts in Malaysia (6,744 cases), followed by Sarawak (2,340 cases), Pulau Pinang (1754 cases), Kelantan (1,620 cases), Kedah (623 cases), Perlis (554 cases) and Selangor (510 cases).

### Correlations of stroke-related Google Trends search queries

Table 2 exhibits correlations between stroke related Google Trends search queries. Stroke symptoms and signs and risk factors were the most searched stroke-related terms in the population. Most stroke-related search queries showed positive correlations with statistical significance (P < 0.05). Across all search queries, “stroke and weakness” showed the strongest positive relationship (r = 0.851, P = 0.014) followed by the risk factor “stroke and family” (r = 0.401, P < 0.001).

**Table 2:** Correlations of stroke-related Google Trends search queries.

Stroke-related hit search volume	Pearson’s correlation coefficient *(r)*	P-value (two tails)
**Disease overview**		
Stroke and brain	0.251	0.001**
Stroke and ischemic	− 0.002	0.980
Stroke and hemorrhagic	− 0.085	0.255
**Symptoms and signs**		
Stroke and symptoms	0.111	0.145
Stroke and headache	0.333	< 0.001**
Stroke and nausea	0.200	0.007**
Stroke and vomiting	0.322	< 0.001**
Stroke and dizziness	0.279	< 0.001**
Stroke and confusion	0.152	0.039*
Stroke and signs	0.161	0.034*
Stroke and weakness	0.851	0.014*
Stroke and speech	0.240	0.001**
**Risk factors**		
Stroke and family	0.401	< 0.001**
Stroke and diabetes	0.355	< 0.001**
Stroke and hypertension	0.577	< 0.001**
Stroke and hypercholesterolemia	0.081	0.275
Stroke and obesity	0.260	< 0.001**
Stroke and smoking	0.272	< 0.001**
Stroke and alcohol	0.346	< 0.001**
**Treatment and prevention**		
Stroke and treatment	0.024	0.744
Stroke and prevention	− 0.012	0.870

*Denotes statistical significance at P < 0.05.

**Denotes statistical significance at P < 0.01.

### Correlations of stroke Google Trends search query and stroke counts

Most states in Malaysia showed statistical significance between ‘stroke’ Google Trends search query with actual counts of stroke. From the countrywide perspective, Malaysia showed a statistically significant negative correlation between ‘stroke’ search query and actual counts data. With the exception of Pulau Pinang and Sarawak that showed a statistically significant positive correlation between ‘stroke’ search query and actual counts data, the remaining states of Perlis, Terengganu, Selangor, Kedah and Sabah showed statistical significance with negative correlations (Table 3).

**Table 3:** Correlations between stroke-related search query and actual stroke counts data.

Actual stroke counts	Stroke search query (*r*)	Actual stroke counts	Stroke search query (*r*)
Perlis	− 0.237*	Pulau Pinang	0.325*
Kelantan	− 0.185	Perak	0.023
Terengganu	− 0.405**	Selangor	− 0.238*
Sarawak	0.766**	Kedah	− 0.521**
Sabah	− 0.382**	MALAYSIA	− 0.835*

Data was mined since 2012 till 31st March 2019 for compatibility with official stroke count registry data. Official count data was retrieved with permissions from NNEUR Malaysia. Malaysia’s stroke count data included nine states (excluded Federal Territories of Kuala Lumpur and Putrajaya, Negeri Sembilan, Johor, Melaka and Pahang due to minimal or unavailability of data for inclusion into analysis). Most correlations were statistically significant yielding evidence that online HISB follows actual count data for further selection into forecasting model.

*Denotes statistical significance at P < 0.05.

**Denotes statistical significance at P < 0.01.

### Forecasting model of stroke in Malaysia

**Figure 5:**
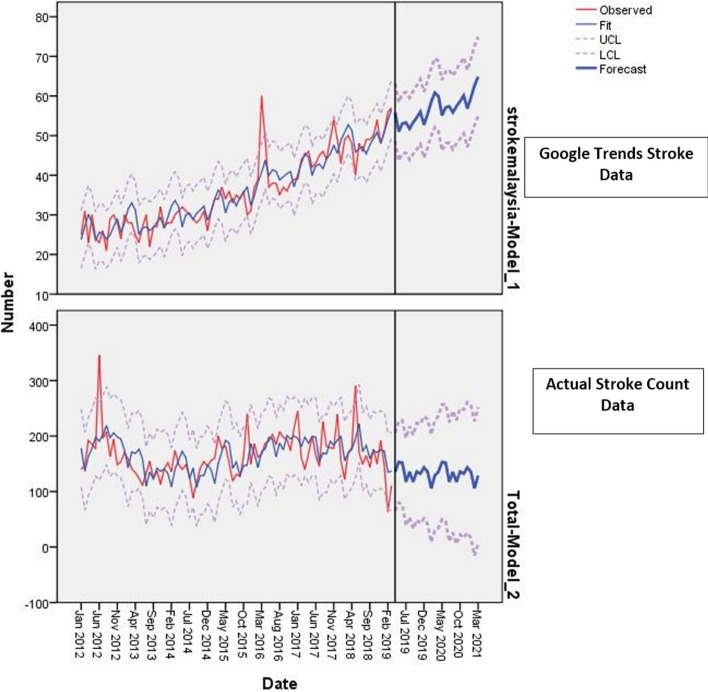
Stroke forecasted model for Malaysia. Forecasted Time Series Modeler was yielded in IBM SPSS Statistics version 22.0^36^.

Figure 5 shows an estimated forecasting model of stroke in Malaysia. The initial correlograms showed that degradation of points in series of time lags to near-zero was slow, suggesting that the data was at non-stationary. We subsequently confirmed stationary based on unit-root tests. The Augmented Dickey Fuller test showed non-statistical significance (P = 0.722), while the Kwiatkowski-Philips-Schmidt-Shin (KPSS) test was statistically significant (P = 0.001), indicating the presence of non-stationary, thus subjecting our model to exponential smoothing. The yielded forecasted model using Winters additive method was statistically significant (P = 0.001), accounting for 62.7% of the total variance explained. The multi-fitted data within the 95% confidence interval showed that ‘stroke’ Google Trends search query would continue to rise but the incidence of stroke may decrease slightly or reach a plateau within the next 3 years (Fig. 5).

## Discussion

This countrywide ecological correlation and time series study utilized the combination of ‘digital epidemiology’ through novel data stream (Google Trends internet data) and ‘classical epidemiology’ of surveillance count data through disease registry that was explicitly aimed to nurture a comprehensive population health-forecasting model of stroke in Malaysia. With rising stroke incidence, we set to address the Malaysian populations’ HISB of stroke in real-time situations, how these behaviors were changing over time with weather variations and geographic gradients, and how would Malaysians be impacted by the current stroke scenario in the future. The trends and patterns yielded in this preliminary spatial epidemiological and time series analytical approach from the Malaysian perspective would set the direction of public health policy preventive measures and tertiary level management guidelines for stroke in the country.

We observed one significant peak of hit searches in 2016. The relatively high search volumes of ‘stroke’ in 2016 could be attributed to the initiation of massive rigorous campaigns and interventions at the hospital and community level nationwide. In 2015, stroke emerged as the second highest non-communicable disease afflicting Malaysians. Malaysia’s leading efforts in combating stroke was recognized by the World Stroke Organization in 2016 when the country’s sole rehabilitation hospital was awarded with the best institutional campaigner to prevent stroke in the low and middle income country category^51^. From the public health perspective, advocates called upon immediate unification of various stakeholders from the government, private and non-governmental organizations to integrate the nationwide hypertension campaign called the “The Morning Hype Campaign” with the “My Stroke Story Photo Exhibition Campaign,” the largest ever representation that involved thirty one stroke survivors who were empowered to submit their photo stories depicting their personal journeys of stroke survival with the desire to live life to the fullest^52^. A touching phenomenon that grabbed media attention in 2016 was the news depicting a Malaysian suffering stroke in London and the family being hit with an excruciatingly high hospital bill, halting further treatment for stroke. Malaysians’ emotions were triggered and an online fund raising campaign was launched to allow fundraisers to channel donations and to follow the health progress of the stroke survivor^53^. These phenomena may have triggered the spike of multiple hit searches of stroke in Google across Malaysia in 2016.

Over an 18-year period, we observed that populations’ HISB of stroke showed a cyclical pattern within a 2-year interval, and subsequently extended to a seasonality trend over the recent years (as evident from Fig. 1). As borderless internet connectivity allows accessibility across all regions in Malaysia with the emergence of Internet of Things (IoTs), the cyclical pattern data yielded through the trend series analysis could be attributed to immediate HISB by stroke afflicted patients, patients’ relatives, family members, colleagues or friends to explore further information about stroke. Google has acknowledged the significance of online health searches and has prioritized the delivery of medically accurate and reliable information^30^. People searching for information on stroke and their outcomes may do so at the time they are experiencing symptoms and may believe that information provided by Google is accurate for the next course of action. Two possible postulations could be derived from the temporal patterns exhibited in our trend analyses. The first is that people may search for symptoms at the time they are experiencing some discomfort such as limb weakness or slurred speech during the onset of stroke or transient ischemic attack. Such searches could be accomplished by the patients themselves at the early onset of symptoms or by their representatives when their clinical conditions deteriorate further. Secondly, seasonality patterns that could extend over months or years could be attributed by searches accomplished by post-stroke survivors to explore disease prognosis, quality of life, disabilities, treatment strategies and cure. Searches at this period of time could also be conducted by patients’ family members, relatives, friends or colleagues to provide social and functional support in view of the debilitating nature of stroke that impairs activities of daily living (ADL) in post-stroke survivors. These situations may have catalyzed periodic ups and downs of ‘stroke’ hit searches frequently via Google Trends. These consistencies were observed in passively generated search queries from Google Trends that have evaluated seasonal patterns in HISB for a variety of non-communicable diseases^2,54–56^.

HISB showed variations between months and weather. We observed greater peaks of hit search volumes between November and April annually which was parallel with the Northeast Monsoon weather, affirming that a causal link may exists between stroke related information-seeking behaviors mediated by higher incidence of stroke during Northeast Monsoon (6,155 cases) as compared to Southwest Monsoon (5,878 cases). Interestingly, the links between HISB and incidence of stroke during Northeast Monsoon were consistent with the geo-spatial distribution of the yielded choropleth maps. Regions affected during this weather season were the East Coast Region and the Northern Region of Peninsular Malaysia. “Red alerts” were conveyed through the distribution maps exhibiting that the states involved in the two regions, namely Kelantan, Terengganu and Perlis were highly prevalent in terms of stroke incidence and stroke search queries in the country. Previous state-specific study showed that Terengganu had relatively high number of stroke cases^33^.

The current study was the first from the Asian perspective that has offered triple anticipated relationships in a spatial epidemiological analysis, showing consistencies between HISB and actual stroke counts data with month, weather and geographical variations in the country. Although these findings were consistent with previous studies that explored HISB from a variety of non-communicable diseases through an ecological perspective^33, 54–56^, these studies were limited with only two associations; the relationships between online HISB either with incidence of the disease or seasonal variations. The linkage of these attributes could not be speculated with the pattern of seasonal variations and geographical distribution coherently. Substantial amount of literature have found considerable amount of evidence that meteorological, temperature or weather variations pose greater risk for the occurrence of stroke^17–19, 57–59^. Much specifically, the seasonal variations of stroke were more likely to be attributed during colder months^60–65^. These trends were consistent with the findings of our current study that stroke incidence, coupled with high HISB were more prevalent during the colder Northeast Monsoon season. A plausibility of such association could be attributed when seasonal changes occur from warmer to cooler temperatures, causing increased blood viscosities or vasoconstriction, a major predictor of stroke^59,64^.

Brigo and colleagues postulated that people with chronic health conditions will frequently use search engines to look for terms related to their disease definitions, etiologies, risk factors, symptoms, treatment and prevention strategies^66^. Our findings were in line with this hypothetical consideration as stroke related Google Trends search queries showed positive correlations with disease pathology, risk factors, symptoms, signs, treatment and prevention. Similar consistencies were observed in online search queries of other diseases or health conditions namely status epilepticus^7^, multiple sclerosis^6^ and systemic lupus erythematous^8^. We also found correlations between HISB and actual stroke incidence across states and countrywide estimate. Although being statistically significant, most states and countrywide associations showed negative correlations between HISB and actual stroke incidence. A plausible explanation of such scenario could be attributed to the nature of the disease or health-related states that are being studied, as the correlation impact of non-communicable diseases are highly complex to decipher due to a number of environmental and lifestyle factors which directly affects the disease states that need to be controlled, such as geography, ethnicity, physical activity, eating habits and social interactions. Although online search queries rise, knowledge of stroke may be improved, lifestyle behaviors could mediate a bidirectional effect of socio-economic status and health. The geographical setting of certain states which are lower in socio-economic status may catalyze a weaker motivation and inadequate resources to maintain a healthy lifestyle. This theoretical model was advocated by Wang & Geng^67^. We also took note of region-specific estimates that were collectively occupied by certain states. The HISB seemed to correlate well with actual stroke cases across regions but correlation of HISB and state-specific counts showed some inconsistencies as discussed earlier. Similar finding was observed in a previous study from the USA^29^. Plausible explanations include: (1) state-specific data captured from the registry dataset that was used for comparison by itself was estimated to be limited; (2) when corresponding to regions, states within the particular region are bulked together, yet states with higher socio-economic status or urban areas have better internet penetration, giving rise to greater search queries and yielding positive relationships with actual stroke cases; and (3) geographic differences (either state or region level) on actual stroke risk factors such as ethnicity, diet, obesity, diabetes mellitus or socio-economic status may serve as surrogate markers for greater internet search interests among the population at risks^29^.

For the first time, we incorporated spatial epidemiology with time series analytics by the utilization of both novel internet data streams and conventional surveillance data of non-communicable diseases. We forecasted a combined impact model that predicted Malaysia’s Stroke 2.0 of HISB and incidence of stroke for the next 3 years. The yielded forecasted model found that, as HISB of stroke continue to rise, the incidence of stroke may slightly decrease or reach a plateau over the next 3 years. Since the spurious peak of stroke searches in 2016 and coupled with ongoing rigorous stroke campaigns, we believed that people tend to explore more about stroke online consistently, thus gaining appropriate up-to-date knowledge on the treatment, control and early prevention of stroke. This could be the reason on why actual stroke cases may have appeared stationary over the subsequent years, yet may be reaching a plateau phase or projected to have a reduced incidence over the next 3 years in our forecasted model. It is giving an important impression that as people explore more information about stroke on the internet, they tend to improve their knowledge and understanding of stroke, succinctly triggering their self-care efforts and control measures to prevent themselves from being afflicted with stroke. We recommend an urgent need for this promising observation through robust analytics and study designs in the near future to test possible variables that may influence such observations. We believe that internet resources have enhanced stroke knowledge, and coupled with efforts of stroke advocates who are currently drafting policy implementations for a paradigm shift of stroke care reform from the vertical to horizontal approaches of prevention strategies through campaigns, community screenings and surveillance efforts may have predicted such observations in the forecasted model.

## Public health implications

Internet data analytics is real-time as compared to conventional surveillance or registry data. This tackles the issue of delayed data collection, analyses, forecasting and interpretation of yielded evidence to inform urgent public health policy. Our analysis identified geographic variations of stroke HISB and actual stroke counts across different states in Malaysia. This approach provides a metric to evaluate health disparities among populations at the national level, informing public health practitioners and advocates in the country to direct community health programs and interventions using targeted approach, such as accelerating stroke risk-factor prevention programs and education measures in disproportionately affected states. Temporal trends from query volumes coupled with their geographic distribution and searches could yield a quantifiable and valuable measure of public attention information needs of stroke. The current results that utilized internet data analytics integrated with conventional registry data would catalyze great opportunities for public health agencies to disseminate health information rapidly and efficiently at a cost-effective pace, provided reliable news are shared to the population. It would be timely to see the acceleration of public health informatics applications in the current sense, where new technology explosion within the population through Google Trends could be used as a proxy for proper diffusion strategies based on health education messages, thus filling translational gap between best evidence and practice. Stakeholders from the public health domain could leverage on these new technologies and information overload to plan proper communication strategies for the prevention of stroke.

## Study strengths and limitations

The current study which used time series analytics through novel internet data streams, (conceptualized as digital epidemiology through the application of infodemiology and infoveillance methodologies) has offset several disadvantages faced by conventional epidemiological approaches. Digital epidemiology provides real-time information of population’s HISB at the national level. Paired with spatial epidemiological approaches, disease states and risk factors could be detected in high risk areas or regions for quick interventions. The approach is cost-effective and quick to be carried out to notify public health advocates for rapid policy drafting and implementations.

Internet data may have certain limitations that need to be cautioned during interpretation. The first is ambiguity of search keywords as Google Trends monitors only queries carried out in Google search engine. The search terms may not be proxy to individuals with stroke or high risk stroke as academics or professionals who are just interested or curious may provide search hits. The anonymity of Google Trends data limits the exploration of stroke HISB across specific demographics, subpopulations and disparities among populations. This is important as the incidence of stroke is stratified across age, ethnicity, gender and socio-economic characteristics^26^. Understanding local HISB of stroke is crucial, but Google Trends data are not available for geographical areas smaller than state or city/town level based on yielded search volumes. Google Trends eliminates repeated queries from the same user over a short period of time to reduce counts of continued searching, and uses a certain threshold of traffic volume so that the very new search terms are assigned to a value of zero, but this could change rapidly. As such, the data may not be independently verified or reliable and investigators have limited control over the data, making quality control difficult.

With the revolution of big public health data, the most popular tool for analyzing HISB using web-based data till date is Google Trends^4^. Online search traffic data was recommended as a good analyzer for internet behavior, and Google Trends has acted as a reliable tool for predicting changes in human behavior; subjected to careful selection of searched terms^4^. With the selection of valid search terms, Google data can accurately measure population’s interest and behavior^68^. As we explored and forecasted a particular disease attribute (in this case “stroke”), the search terms and queries will be constant over time. With such valid and consistent terms used to explore disease attributes (e.g. symptoms and signs, risk factors, treatment, etc.), the search terms and analysis are replicable for future research, thus ensures reliability. Moreover, our search terms exploration technique was based on the validated model as proposed by Mavragani & Ochoa^13^.

Due to the nature of the ecological-correlation study design, the results of our study may be subjected to ecological fallacy as there may be mismatch of drawing conclusions about individual-level stroke epidemiological associations from a group-level data. However, it is a unique and a more appropriate study design to explore trends and patterns for observing correlations of exposures at the population level in exploring a particular disease or public health phenomenon. The current study may be subjected to “mixing” as geographical variations may suffer migrations of population within states, thus diluting differences between groups in our study population. To be consistent with epidemiological concepts in determining disease distribution and determinants, future research using Google Trends data should incorporate individual tracing when users are logged in to their accounts, thus enabling user characteristics retrieval and analyses such as age, gender and ethnicity. The intent of the study would catalyze more meaningful interpretations based on disease risk stratifications of stroke. Such opportunity and usefulness of Google Trends data should be maximized to facilitate public health interventions, health education and promotions, but should be cautioned of use with relevant privacy settings assured.

## Conclusion

The current study has provided insights on trends of stroke HISB from internet data that showed possible associations with weather and geographical variations through time series analytics and spatial epidemiology approaches. Search queries were correlated positively with disease characteristics but negatively with actual stroke counts data. Our forecasted model showed that HISB will continue to rise but stroke incidence may reach a plateau within the next 3 years. The current study has offered new real-time surveillance tool and approaches to alert public health systems and policy makers for planning appropriate resources towards stroke detection and prevention in the country. Future studies should validate internet based data with external datasets for reliable use of such approaches.

## Data Availability

The dataset of NNEUR retrieved and analyzed during this study is not available publicly due to local ethics regulation and could be obtained via written permissions to NNEUR and the Director General of Health, Ministry of Health Malaysia. Internet dataset is publicly available via Google Trends website domain (https://trends.google.com/trends/explore).

## References

[1] Xing L (2020). Epidemiology of stroke in urban northeast China: a population based study 2018–2019. Int. J. Stroke.

[2] Kumar, N., Pandey, A., Garg, N., Sampene. E. & Lavie, C. J. An analysis of the online search trends. Seasonal and geographic patterns in seeking cardiovascular health information. *Mayo Clin. Proc.* **93**, 1185–1190 (2018). 10.1016/j.mayocp.2018.07.011 PMC7089782 30193672

[3] Aragon, T. J. *Population Health Data Science with R*. https://bookdown.org/taragonmd/phds/ (2019).

[4] Mavragani A, Ochoa G, Tsagarakis KP (2018). Assessing the methods, tools and statistical approaches in Google Trends research: systematic review. J. Med. Internet Res..

[5] Ayers JW, Althouse BM, Dredze M (2014). Could behavioral medicine lead the web data revolution?. JAMA.

[6] Bragazzi NL (2013). Infodemiology and infoveillance of multiple sclerosis in Italy. Mult. Scler. Int..

[7] Bragazzi NL, Bacigaluppi S, Robba C, Nardone R, Trinka E, Brigo F (2016). Infodemiology of status epilepticus: a systematic validation of the Google Trends-based search queries. Epilepsy Behav..

[8] Radin M, Sciascia S (2017). Infodemiology of systemic lupus erythematous using Google Trends. Lupus.

[9] Arora VS, Stuckler D, McKee M (2016). Tracking search engine queries for suicide in the United Kingdom, 2004–2013. Public Health.

[10] Bragazzi NL (2013). A Google Trends-based approach for monitoring NSSI. Psychol. Res. Behav. Manag..

[11] Foroughi F, Lam AK, Lim MS, Saremi N, Ahmadvand A (2016). “Googling” for cancer: An infodemiological assessment of online search interests in Australia, Canada, New Zealand, the United Kingdom and the United States. JMIR Cancer.

[12] Cavazos-Rehg PA (2015). Monitoring of non-cigarette tobacco use using Google Trends. Tob. Control.

[13] Mavragani A, Ochoa G (2018). Forecasting AIDS prevalence in the United States using online search traffic data. J. Big Data.

[14] Alicino C (2015). Assessing Ebola-related web search behaviour: insights and implications from an analytical study of Google Trends-based query volumes. Infect. Dis. Poverty.

[15] Cho S (2013). Correlation between national influenza surveillance data and Google Trends in South Korea. PLoS ONE.

[16] Domnich A, Panatto D, Signori A, Lai PL, Gasparini R, Amicizia D (2015). Age-related differences in the accuracy of web query-based predictions of influenza-like illness. PLoS ONE.

[17] Low RB, Bielory L, Qureshi AI, Dunn V, Stuhlmiller DFE, Dickey DA (2006). The relation of stroke admissions to recent weather, airborne allergens, air pollution, seasons, upper respiratory infections, and asthma incidence, September 11, 2001, and day of the week. Stroke.

[18] Wang XY, Barnett AG, Hu W, Tong S (2009). Temperature variation and emergency hospital admissions for stroke in Brisbane, Australia, 1996–2005. Int. J. Biometeorol..

[19] Goggins WB, Woo J, Ho S, Chan EYY, Chau PH (2012). Weather, season, and daily stroke admissions in Hong Kong. Int J Biometeorol.

[20] Feigin VL (2015). Update on the global burden of ischemic and hemorrhagic stroke in 1990–2013: the GBD 2013 study. Neuroepidemiology.

[21] Feigin VL, Norrving B, Mensah GA (2017). Global burden of stroke. Circ. Res..

[22] GBD 2016 Stroke Collaborators. Global, regional, and national burden of stroke, 1990–2016: A systematic analysis for the Global Burden of Disease Study 2016. *Lancet Neurol.* **18**, 439–458 (2019). 10.1016/S1474-4422(19)30034-1 PMC6494974 30871944

[23] Avan A (2019). Socioeconomic status and stroke incidence, prevalence, mortality, and worldwide burden: an ecological analysis from the Global Burden of Disease Study 2017. BMC Med..

[24] Gorelick PB (2019). The global burden of stroke: persistent and disabling. Lancet Neurol..

[25] Kooi CW, Peng HC, Aziz ZA, Looi I (2016). A review of stroke research in Malaysia from 2000–2014. Med. J. Malaysia.

[26] Aziz, Z. A. & Sidek, N. N. Annual Report of the Malaysian Stroke Registry 2009–2016. *In Kuala Terengganu Clinical Research Centre* (2017).

[27] Institute for Health Metrics and Evaluation. *Statistics Data* https://www.healthdata.org/malaysia (2019).

[28] Lee YY, Shafie AA, Sidek NN, Aziz ZA (2017). Economic burden of stroke in Malaysia: results from National Neurology Registry. J. Neurol. Sci..

[29] Walcott BP, Nahed BV, Kahle KT, Redjal N, Coumans JV (2011). Determination of geographic variance in stroke prevalence using internet search engine analytics. Neurosurg Focus.

[30] Senecal C, Widmer RJ, Lerman LO, Lerman A (2018). Association of search engine queries for chest pain with coronary heart disease epidemiology. JAMA Cardiol.

[31] Eysenbach G (2009). Infodemiology and infoveillance: framework for an emerging set of public health informatics methods to analyze search, communication and publication behavior on the Internet. J. Med. Internet Res..

[32] Google Trends https://trends.google.com/trends/explore (2019)

[33] Aziz ZA (2015). Acute Stroke Registry Malaysia, 2010–2014: Results from the National Neurology Registry. J Stroke Cerebrovasc Dis.

[34] Mavragani A, Ochoa G (2019). Google Trends in infodemiology and infoveillance: methodology framework. JMIR Public Health Surveill..

[35] R Core Team. R: A language and environment for statistical computing. R Foundation for Statistical Computing, Vienna, Austria, https://www.R-project.org/ (2018).

[36] IBM Corp. IBM SPSS Statistics for Windows, Version 22.0. Armonk, NY: IBM Corp, (2013).

[37] Wessa, P. Multiple Regression (v1.0.6) in free statistics software (v1.1.23-r7). Office for research development and education https://www.wessa.net/rwasp_correlation.wasp/ (2019).

[38] Global Administrative Dataset, Center of Spatial Sciences https://gadm.org/download_country_v3.html (2019).

[39] Gogtay NJ, Thatte UM (2017). Principles of correlation analysis. J. Assoc. Physicians India.

[40] Schober P, Boer C, Schwarte LA (2018). Correlation coefficients: appropriate use and interpretation. Anesth. Analg..

[41] SPSS Quick Tutorials. Pearson correlations—quick introduction https://www.spss-tutorials.com/pearson-correlation-coefficient/ (2020).

[42] Robinson WS (2009). Ecological correlations and the behavior of individuals. Int. J. Epidemiol..

[43] BMJ. Epidemiology for the uninitiated. Chapter 6: Ecological studies. https://www.bmj.com/about-bmj/resources-readers/publications/epidemiology-uninitiated/6-ecological-studies (2020).

[44] Hyndman, R. J. & Athanasopoulos, G. *Forecasting: Principles and Practice*, 2nd edition, 2018*. *OTexts: Melbourne, Australia OTexts.com/fpp2 (2020).

[45] Amini P (2018). Modelling the frequency of depression using Holt-Winters exponential smoothing method. J. Clin. Diagn. Res..

[46] Trull O, Garcia-Diaz JC, Troncoso A (2020). Initialization methods for multiple seasonal Holt-Winters forecasting models. Mathematics.

[47] Prajakta SK (2004). Time series forecasting using holt-winters exponential smoothing. Kanwal Rekhi School Inf. Technol..

[48] Pan R (2010). Holt-Winters Exponential Smoothing.

[49] Box GE, Jenkins GM, Reinsel GC, Ljung GM (2015). Time series analysis: forecasting and control.

[50] Winters PR (1960). Forecasting sales by exponentially weighted moving averages. Management.

[51] Malaysia Healthcare Travel Council https://www.mhtc.org.my/mhtc/2017/03/01/2016-world-stroke-campaign-award/ (2017).

[52] Press-reader https://www.pressreader.com/malaysia/the-borneo-post/20161024/283137133328910 (2016).

[53] New Straits Times https://www.nst.com.my/news/2016/07/161504/malaysian-suffers-stroke-london-family-hit-%C2%A346000-hospital-bill (2016).

[54] Ayers JW, Althouse BM, Allem JP, Rosenquist JN, Ford DE (2013). Seasonality in seeking mental health information on Google. Am. J. Prev. Med..

[55] Ingram DG, Plante DT (2013). Seasonal trends in restless legs symptomatology: evidence from Internet search query data. Sleep Med..

[56] Madden KM (2017). The seasonal periodicity of healthy contemplations about exercise and weight loss: ecological correlational study. JMIR Public Health Surveill..

[57] Berginer VM, Goldsmith J, Batz U, Vardi H, Shapiro Y (1989). Clustering of strokes in association with meteorologic factors in the Negev Desert of Israel: 1981–1983. Stroke.

[58] Kyobutungi C, Grau A, Stieglbauer G, Becher H (2005). Absolute temperature, temperature changes and stroke risk: a case crossover study. Eur. J. Epidemiol..

[59] Chen R (2013). Both low and high temperature may increase the risk of stroke mortality. Neurology.

[60] Jakovljevic D (1996). Seasonal variation in the occurrence of stroke in a Finnish adult population. The FINMONICA Stroke Register. Finnish Monitoring Trends and Determinants in Cardiovascular Disease. Stroke.

[61] Spengos K (2003). Diurnal and seasonal variation of stroke incidence in patients with cardioembolic stroke due to atrial fibrillation. Neuroepidemiology.

[62] Klimaszewska K (2007). Seasonal variation in ischaemic stroke frequency in Podlaskie Province by season. Adv. Med. Sci..

[63] Christensen AL (2012). Seasonality, incidence and prognosis in atrial fibrillation and stroke in Denmark and New Zealand. BMJ Open.

[64] Rakers F (2016). Rapid weather changes are associated with increased ischemic stroke risk: a case-crossover study. Eur. J. Epidemiol..

[65] Lichtman JH, Leifheit-Limson EC, Jones SB, Wang Y, Goldstein LB (2016). Average temperature, diurnal temperature variation and stroke hospitalizations. J. Stroke Cerebrovasc. Dis..

[66] Brigo F (2014). Web search behavior for multiple sclerosis: an infodemiological study. Mult. Scler. Relat. Disord..

[67] Wang J, Geng L (2019). Effects of socioeconomic status on physical and psychological health: lifestyle as a mediator. Int. J. Environ. Res. Public Health.

[68] Scharkow M, Vogelgesang J (2011). Measuring the public agenda using search engine queries. Int. J. Public Opin. Res..

